# Real-World Data on Outcomes in Metastatic Castrate-Resistant Prostate Cancer Patients Treated With Abiraterone or Enzalutamide: A Regional Experience

**DOI:** 10.3389/fonc.2021.656146

**Published:** 2021-06-18

**Authors:** Rachel Raju, Arvind Sahu, Myron Klevansky, Javier Torres

**Affiliations:** ^1^ Department of Oncology, Goulburn Valley Health, Shepparton, VIC, Australia; ^2^ Department of Rural Health, Faculty of Medicine, Dentistry and Health Sciences, University of Melbourne, Shepparton, VIC, Australia

**Keywords:** real world, regional, metastatic prostate cancer, abiraterone, enzalutamide

## Abstract

**Background:**

Both abiraterone and enzalutamide have shown to improve overall survival (OS), progression-free survival (PFS) and prostate-specific antigen (PSA) response in patients with metastatic castration-resistant prostate cancer (mCRPC) regardless of previous treatment with chemotherapy (COU-AA301^1^, COU-AA302^2^, AFFIRM^3^ and PREVAIL^4^). The data regarding the impact of these treatments in the real world setting is scarce. This study assessed the real world survival and disease outcomes in mCRPC patients in a regional health service in Victoria with the use of abiraterone and enzalutamide.

**Methods:**

This retrospective clinical audit included 75 patients with diagnosis of mCRPC treated with either abiraterone or enzalutamide between January 1, 2014, and December 31, 2019, at Goulburn Valley Health. Patients were stratified according to the drug received, Eastern Cooperative Oncology Group (ECOG) performance status, Gleason score, burden of disease at diagnosis, presence of visceral metastases and use of previous chemotherapy. The primary end point was PSA response (defined as a reduction in the PSA level from baseline by 50% or more). The secondary outcomes were PSA PFS, radiographic PFS, and OS.

**Results:**

Thirty-seven patients received enzalutamide, and the other 38 received abiraterone. Only 20% of patients in either group had visceral metastases. 32% of patients receiving enzalutamide had a high burden of disease, compared to 53% receiving abiraterone. 38% of patients in the enzalutamide group and 53% in the abiraterone group had received prior chemotherapy. PSA response rates were higher in the enzalutamide group than abiraterone group (70.3% vs 37.8%). Both PSA and radiographic PFS were longer in the enzalutamide group than abiraterone group; 7 months vs 5 months for both end points. OS was also found to be longer in patients receiving enzalutamide; 30 months compared to only 13 months in patients receiving abiraterone.

**Conclusion:**

Both abiraterone and enzalutamide have shown to result in significant PSA response rates, as well as PFS and OS benefit in mCRPC patients in the real world setting. The difference in responses and survival benefit are probably impacted by the unbalanced burden of disease.

## Introduction

Both abiraterone and enzalutamide are current standard of care treatments for patients with metastatic castrate-resistant prostate cancer (mCRPC), and are widely used in clinical practice. These agents have shown to improve overall survival (OS), prostate-specific antigen (PSA) response, and radiographic and PSA progression-free survival (PFS) in mCRPC patients regardless of prior chemotherapy use, as reflected in large phase III clinical trials; COU-AA301 (1), COU-AA302 (2), AFFIRM (3), and PREVAIL (4). In the COU trials, abiraterone with prednisolone compared to placebo and prednisolone resulted in a PFS and OS benefit in mCRPC patients who had prior docetaxel chemotherapy, but only a PFS benefit was demonstrated in chemotherapy naïve mCRPC patients (1, 2). AFFIRM and PREVAIL demonstrated a PFS and OS benefit of enzalutamide over placebo in mCRPC patients, with or without prior use of docetaxel (3, 4). Quality of life improvement has also been shown in these studies with abiraterone and enzalutamide, with reduction in time to first skeletal related event and improved pain management in this group of patients. This quality of life data is especially important in patients with metastatic prostate cancer, as bony metastases can be extensive and symptomatic, and can result in acute neurological sequelae such as cord compression and cauda equina syndrome.

The decision of choosing one agent over the other is individualised, as to date there are no prospective studies evaluating the sequencing of abiraterone and enzalutamide. A randomised phase II sequencing trial involving 202 chemotherapy naïve patients with mCRPC assigned to abiraterone plus prednisolone or enzalutamide with crossover allowed, demonstrated no significant difference between first-line abiraterone and first-line enzalutamide in terms of time to PSA progression [median 11·2 vs 10·2 months, Hazard ratio (HR) = 0·95, 95% CI 0·66–1·36, p = 0·78]. The abiraterone-first arm had longer time from start of first-line therapy to second PSA progression (median 28·4 months vs 14·2 months, HR = 0·65, 95% CI 0·36–1·17, p = 0·15) and higher second PSA responses. However, there was no statistically significant difference in OS between the two arms (5). A meta-analysis aimed at comparing the efficacies between abiraterone and enzalutamide in mCRPC patients, using pooled results of 19 studies, found that treatment with first-line enzalutamide was associated with an increase in median OS of 5.9 months (HR 0.81, p<0.001) and an increase in median PFS of 8.3 months (HR = 0.47, p<0.001) compared to abiraterone in the pre-docetaxel mCRPC setting. In the post-docetaxel setting, enzalutamide was shown to have a small but statistically significant (especially after adjusting for baseline Gleason score) advantage over abiraterone with respect to PFS (6).

Prospective trial validation comparing efficacies of one androgen receptor blocker to the other, however, is lacking. The PFS and OS outcomes of abiraterone and enzalutamide in clinical practice especially in regional health centres in Australia have not been studied. Variability in drug tolerability due to differences in ECOG performance status and comorbidities, as well as compliance in the real world population can affect outcomes. This retrospective real world study assessed the PSA response, PFS and OS outcomes in mCRPC patients on enzalutamide and abiraterone in a regional health service in Victoria (Australia).

## Methods

### Participants and Data Definitions

Patients with the diagnosis of mCRPC treated with either abiraterone or enzalutamide between the period January 1, 2014, and December 31, 2019, at Goulburn Valley Health were included in this retrospective audit. Any prior treatment including chemotherapy was allowed. Individual patient electronic records were reviewed and data recorded on to an Excel spreadsheet. The demographic data and baseline patient and tumour characteristics were collected from the electronic medical record system. Patient characteristics including age and ECOG performance status, as well as tumour characteristics including Gleason score, burden of disease at diagnosis (high volume defined as presence of visceral metastases and/or four or more bony metastases with one or more beyond vertebral body and pelvis), presence of visceral metastasis, prior systemic therapies were recorded from patient hospital files and hospital electronic medical records. Radiological and biochemical response to treatment, as well as tolerability was recorded.

The primary outcome was PSA response rates. The secondary outcomes were PSA PFS, radiographic PFS, and OS. PSA response was defined as a reduction in the PSA level from baseline by 50% or more (3). PFS was defined as time from treatment initiation with abiraterone or enzalutamide to disease progression, measured either biochemically *via* PSA readings alone or in combination with radiological staging utilising CT and whole body bone scans. The definition of biochemical disease progression was based on the Prostate Cancer Clinical Trials Working Group (PCWG-3) criteria (7). The definition of radiological progression was based on the WHO criteria in tumour response (8). OS was defined as time from treatment initiation with abiraterone or enzalutamide to time of death of any cause.

### Statistical Analysis

Survival was assessed in using the Kaplan-Meier method, and tested by means of a two-sided log-rank test. A Cox proportional hazards model was used to perform multivariable analysis of various factors affecting OS, including study intervention. All analyses were performed using SPSS Statistics for Windows software, version 26.0 (SPSS, Chicago, IL).

## Results

### Patient and Disease Characteristics

**Figure 1:**
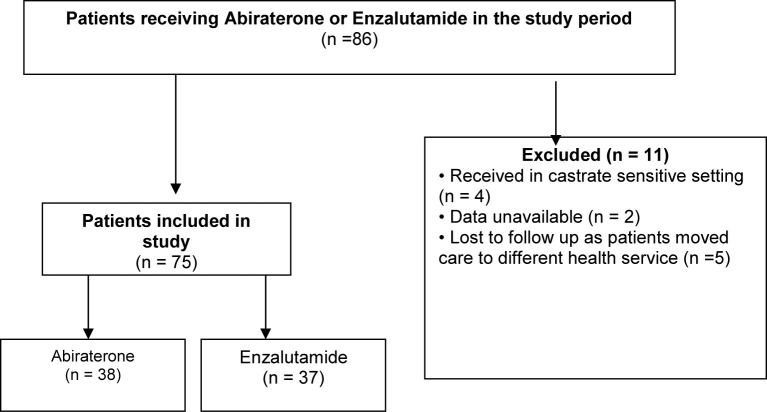
CONSORT diagram.

Information was collected on 86 patients in total, but 11 patients were ultimately excluded due to various reasons **(** **Figure 1** **).** A total of 75 patients were divided into two groups based on whether they received abiraterone or enzalutamide, and stratified according to ECOG performance, Gleason score, burden of disease, presence of visceral metastases and use of previous systemic therapy including chemotherapy and other androgen blockade therapies **(** **Table 1** **)**. Median age was 80 years old (61–94 years old), with most patients having an ECOG performance status of 1 (39%) or 2 (36%). About half of the patients in either group had a Gleason score of at least 8 or above. 55% of patients on abiraterone and 64% on enzalutamide were previously treated with other anti-androgen blockers. Median follow up duration was 37 months.

**Table 1:** Baseline patient and disease characteristics in the abiraterone and enzalutamide groups.

Characteristic	Abiraterone	Enzalutamide
	(N = 38)	(N = 37)
**Age, year**		
* Median	80	80
* Range	61–94	61–94
**ECOG status- no. (%)**		
* 0	1 (2)	1 (3)
* 1	12 (32)	17 (46)
* 2	17 (45)	10 (27)
* 3	8 (21)	9 (24)
**Gleason score (%)**	6 (16)	9 (24)
* 6–7	19 (50)	21 (57)
* 8–10	13 (34)	7 (18)
* Unknown		
Burden of disease* at diagnosis- no. (%)		
* High	20 (53)	12 (32)
* Low	18 (47)	25 (68)
**Visceral metastases- no. (%)**	8 (21)	7 (19)
**Prior systemic treatment- no. (%)**		
* Docetaxel	20 (53)	14 (38)
* Abiraterone	–	2 (5)
* Enzalutamide	9 (24)	–
* Other antiandrogens	21 (55)	24 (64)

*Defined as high volume defined as presence of visceral metastases and/or four or more bony metastases with one or more beyond vertebral body and pelvis.

### Disease and Survival Outcomes

**Figure 2:**
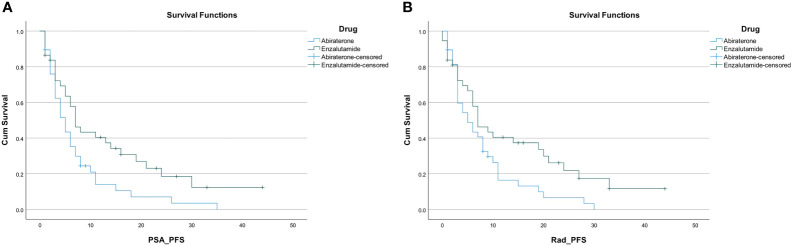
**(A)** PSA progression free survival. **(B)** Radiographic progression free survival.

PSA response occurred in 54% of the entire study population (41 out of 75 patients). A higher proportion of patients in the enzalutamide group had a PSA response; 26 out of 37 patients (70.3%) compared to only 15 out of 38 patients (39.5%) in the abiraterone group. The PSA PFS was 6 months (95% CI, 4.5–7.5) in the entire cohort. Patients on enzalutamide experienced a PSA PFS of 7 months (95% CI, 4.7–9.3), compared to 5 months (95% CI, 3.3–6.7; p=0.022) for patients on abiraterone. Radiographic PFS in the enzalutamide group was 7 months (95% CI, 3.6–10.4) compared to 5 months in the abiraterone group (95% CI, 2.0–8.0; p=0.036) (**Figures 2A, B** **)**.

PSA PFS was found to be 7 months in the subgroup with low burden of disease, and 4 months in the subgroup with high burden of disease. PSA PFS was 7 months in the chemotherapy naïve subgroup, and 4 months in the chemotherapy experienced subgroup. Radiographic PFS was found to be 8 months in those with low burden of disease, and 6 months in those with high burden of disease. Radiographic PFS was 7 months in the chemotherapy naïve group, and 6 months in those who have had prior chemotherapy.

**Figure 3:**
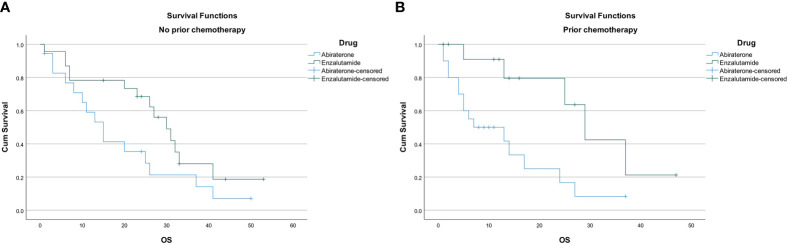
**(A)** Overall survival in chemotherapy naive patients. **(B)** Overall survival in patients with prior chemotherapy use.

Overall survival was 24 months (95% CI, 15.5–32.5) in the entire cohort. Overall survival was found to be longer in patients receiving enzalutamide compared to abiraterone regardless of previous chemotherapy use; 30 months (95% CI, 23.3–36.7) versus 15 months (95% CI, 9.7–20.3; p=0.002) in those who were chemotherapy naïve; and 29 months (95% CI 21.3–36.7) versus 7 months (95% CI, 0–18.5; p=0.002) in those with prior chemotherapy use (**Figures 3A, B**).

On univariate analysis, enzalutamide use (HR 0.405; p value 0.002), dose reduction (HR 1.68; p value 0.05), ECOG performance status <2 (HR 0.71; p value 0.03) and high disease burden (HR 1.56; p value 0.05) had significant association with OS. Only ECOG < 2 (HR 0.66; p value 0.03) showed significant independent effects on survival on multivariate analysis. None of the other factors (age, presence or absence of visceral metastasis, Gleason’s score or prior chemotherapy were associated with impact on OS on both univariate and multivariate analysis.

### Tolerability

No patients were started on upfront dose reductions of either abiraterone or enzalutamide. Dose reductions subsequently occurred in 24% of the entire study population (18 out of 75 patients). A higher proportion of patients receiving enzalutamide required a dose reduction; 13 patients (35%) compared to 5 patients (13%) receiving abiraterone. Dose interruptions or delays occurred in 21% of the entire cohort (16 out of 75 patients); this appeared to be similar; 8 patients (21%) in each of the enzalutamide and abiraterone groups. The most common reasons for dose reductions or delays for patients on enzalutamide were fatigue (8 patients); 2 patients experienced drowsiness and 1 patient’s enzalutamide was ceased after a haemorrhagic stroke. As for abiraterone, liver function test derangement (2 patients), drowsiness (2 patients), fatigue (1 patient) and an unrelated acute medical illness requiring hospital admission (1 patient) were reasons for dose reductions or delays. Reasons for dose reductions or delays in the other patients were not clear from the medical records.

## Discussion

The positive survival outcomes of abiraterone and enzalutamide have long been proven in the mCRPC population in large phase III clinical trials [COU-AA301 (1), COU-AA302 (2), AFFIRM (3) and PREVAIL (4)]. Our study found that despite the variability in both patient and disease factors in the regional Australia real world setting, abiraterone and enzalutamide remain effective treatment options in our clinical practice, and provide a significant survival benefit and disease control in this group of patients with mCRPC.

To date, there is no evidence suggesting one drug is superior to the other in terms of survival outcomes. The four landmark clinical trials in this space (AFFIRM, PREVAIL, COU-AA301 and COU-AA302) demonstrated that the median OS in chemotherapy naïve mCRPC patients was close to 3 years for both enzalutamide and abiraterone; 32.4 months and 34.7 months respectively. PSA response rates were observed to be higher with enzalutamide; 54% versus 38% with abiraterone in the post docetaxel setting. Similarly, in a real world retrospective study conducted in the United Kingdom (9), a greater PSA50 (defined as the percentage of patients who had a PSA decline of at least 50% from baseline) was seen in the enzalutamide group compared to abiraterone group (58% versus 31% p<0.0005), but there was no significant median OS difference between the groups (enzalutamide 13.8 months versus abiraterone 12.5 months p=0.065). Responses on enzalutamide were further supported by a retrospective cohort study (10), that showed a PSA50 of 55% in 931 men with mCRPC on enzalutamide therapy. A meta-analysis (11) demonstrated superiority of enzalutamide over abiraterone in terms of radiographic PFS, time until PSA progression, and PSA response rate in both the pre‐ and post‐docetaxel settings, but again OS did not differ significantly between the two drugs.

In our study, the survival outcomes from enzalutamide appear to match the results from the phase III trials more closely than abiraterone, which is likely due to the unbalanced disease burden and ECOG performance status between the two groups. Specifically, the OS with enzalutamide was about 30 months with or without prior chemotherapy, which is similar to results from AFFIRM and PREVAIL. PSA responses for enzalutamide seen in our study appear to be similar to other retrospective trials mentioned above (9, 10). Patients receiving abiraterone however appeared to have a much poorer survival outcome; only 15 months and 7 months for no chemotherapy and prior chemotherapy respectively. Interestingly, similar to our study, real world studies on abiraterone in mCRPC have showed poorer outcomes than in the COU trials. A Singaporean retrospective audit (12) looking at abiraterone in the real world mCRPC population of 200 patients demonstrated a median OS of 20 months for men who were chemotherapy naïve and only 11.3 months for men who have had prior chemotherapy. The variability in patient population in terms of ECOG performance status and comorbidities in the real world are important factors to consider given the majority of patients with prostate cancer are elderly often with multiple medical problems. These chronic medical issues particularly active cardiovascular comorbidities such as ischemic heart disease, congestive heart failure and strokes can affect the type of anti-cancer therapy these patients with mCRPC receive. Abiraterone is associated with more frequent cardiac events, myocardial infarction, arrythmia and heart failure (13), hence it is usually contraindicated in patients with cardiac comorbidities in particular congestive heart failure. In the phase III trials, the median age was 70 years old and majority of participants (up to 90%) were ECOG 0–1. In contrast, our real world study included patients with a median age of 80 years old, with majority being ECOG 1–2 with 20% of patients being ECOG 3. Compliance rates due to side effect profile and psychosocial factors can also be variable in the real world population, and can ultimately affect survival outcomes (14). As a result, the real world patient population at times are under represented in large phase III clinical trials where disease factors (such as Gleason score, burden of disease, and presence of visceral metastases) are the key differentiating mechanisms affecting outcomes.

One of the limitations to this study is that it was a small single-centre retrospective study. The unbalanced patient and disease characteristics between the two groups likely contributed to the differences in outcomes. There was a higher proportion of patients taking abiraterone who were classified as having high burden of disease, and more patients on abiraterone had prior chemotherapy (docetaxel) use compared to those on enzalutamide. This would suggest that the group taking abiraterone likely had a more aggressive biology of their metastatic prostate cancer, requiring more lines of treatment prior to abiraterone. Majority of patients on abiraterone were ECOG 2 compared to those on enzalutamide who were ECOG 1 and thus more medically fit. The greater proportion of ECOG 1 and 2 patients noted in our audit compared to the COU trials would be consistent with our practice of commencing these less medically fit patients on androgen receptor targeted agents rather than chemotherapy. In the real word setting, mCRPC patients with advanced age and poorer performance status are sequenced to a different anti-androgen rather than chemotherapy on disease progression due to concerns about tolerance. This can have impact on survival outcomes. A subset analysis in Chan et al. of chemotherapy naive patients with an ECOG 2–4 showed a poorer OS and PFS (12). Similarly, Boegemann et al. (15), also showed that poorer ECOG was associated with shorter time to treatment failure.

There has been a few sequencing studies of abiraterone and enzalutamide in the mCRPC space. Khalaf et al. (5) suggest that enzalutamide may be used effectively after abiraterone (rather than vice versa), based on the improved second PSA response and time to second PSA progression. Another single arm, multicentre study (16) included 214 mCRPC patients who commenced on enzatalumide 24 weeks or more after progressing on abiraterone and prednisolone, with or without prior chemotherapy. This study showed a median radiographic PFS of 8.1 months (95% CI: 6.1–8.3) and a median time-to-PSA progression of 5.7 months (95% CI: 5.6–5.8), however the median OS had not been reached. The anti-tumour activity of enzalutamide after abiraterone was further confirmed by Azad et al. (17), demonstrating a median time to PSA progression of 4.63 months (95% CI: 3.11–6.15) and 6.64 months (95% CI: 2.82–10.46), and a median OS of 10.58 months (7.16–14.00) and 8.64 months (6.57–11.71) for both chemotherapy experienced and chemotherapy naïve patients respectively. However, the CARD randomised control trial showed a significantly longer median PFS and OS in mCRPC patients given cabazitaxel compared to those who had abiraterone after enzalutamide or vice versa (18), concluding that in this group of patients if fit enough, further chemotherapy is still the preferred option. In our study, only 9 (24%) of patients in the abiraterone group received previous enzalutamide and 2 (5%) of patients in the enzalutamide group received previous abiraterone; hence making the numbers too small to draw any conclusions.

In terms of tolerability, the REAAcT prospective, real-world study showed grade 3 and 4 adverse events appeared to be similar for both abiraterone and enzalutamide, although fatigue was more commonly reported by patients on enzalutamide compared to those on abiraterone (26% vs 8%). In this study, there was found to be a statistically significant worsening of fatigue for patients on enzalutamide using the FACIT (Functional Assessment of Chronic Illness Therapy Fatigue subscale)-Fatigue score, but not using the other two patient reported outcome instruments. Dose reductions were more common in enzalutamide (16% vs 6%) but dose adjustments and interruptions were similar (19). Similarly, a retrospective cohort study from the British Columbia Cancer Agency looking at abiraterone and enzalutamide in elderly patients with mCRPC showed more patients treated with enzalutamide needed dose reductions due to fatigue (20). We observed in our study that dose reductions were also more frequent for patients on enzalutamide than those on abiraterone, but dose interruptions and delays appeared to be similar in both groups. Similar to previous real world studies, we observed that fatigue was the main reason for dose reductions or delays in the enzalutamide group.

Recently, an electronic CRPC Australian database (ePAD), which is a multi-site, national prospective cohort study, has been commenced to analyse treatment patterns and outcomes from real-world patients with CRPC. Data is being collected regarding baseline patient characteristics, details at diagnosis, pathological characteristics, local treatment and use of androgen deprivation therapy, diagnosis of castration-resistance, prescription of and effectiveness of each systemic therapy and survival (21). This will aim to provide further guidance to Australian medical oncologists when it comes to decision making around systemic treatment selection and rationale for change of treatments in our CRPC patients.

## Conclusion

Both abiraterone and enzalutamide will remain standard of care treatments in Australian men with mCRPC, as the survival and disease control benefits of these agents have continued to be seen in numerous real world studies, consistent with the phase III clinical trials. Although some retrospective studies demonstrate the superior efficacy of enzalutamide over abiraterone, to date very limited prospective trials with head-to-head comparison between these agents exist to adequately support these results. ECOG performance status and to a lesser extent age, which are key variability factors in the real world population do have an impact on survival outcomes. We await data from the Australian ePAD registry to further provide us with real world patient outcomes to support and improve our clinical practice in the mCRPC space.

## Data Availability Statement

The original contributions presented in the study are included in the article/**Supplementary Material**. Further inquiries can be directed to the corresponding author.

## Author Contributions

RR, first author, involved in performing all data collection and preparation of manuscript and poster (eposter) presentations for various conferences AS, second and corresponding author, involved in performing data analysis and review of manuscript and provision of feedback and comments to first author. MK, subsequent author, involved in review of manuscript and provision of feedback. JT, subsequent author, involved in review of manuscript and provision of feedback. All authors contributed to the article and approved the submitted version.

## Conflict of Interest

The authors declare that the research was conducted in the absence of any commercial or financial relationships that could be construed as a potential conflict of interest.
