# Supplementary material for: Real-World Data on Outcomes in Metastatic Castrate-Resistant Prostate Cancer Patients Treated With Abiraterone or Enzalutamide: A Regional Experience
Source: Front Oncol. 2021 Jun 18;11:656146. doi: 10.3389/fonc.2021.656146 (PMC8249852; doi:10.3389/fonc.2021.656146)

***Abiraterone – Enzalutamide Data Sheet Results***

75 patients with metastatic castrate resistant prostate cancer treated with abiraterone or enzalutamide between August, 2013 to Dec, 2019 at GVH.

**Whole cohort results**

- Abiraterone = 38 (Aug, 2013 – Dec 2019)
- Enzalutamide = 37 (July, 2015 to Dec, 2019)
- Median Age = 80 (61-94)
- ECOG
  - 0 = 2 (2.7%)
  - 1 = 29 (38.7%)
  - 2=27 (36.%)
  - 3 = 17 (22.7%)
- Risk Stratification
  - High = 54 (72%)
  - Low = 11 (14.7%)
  - Unknown = 10 (13.3%)
- Burden of Disease
  - High = 32 (42.7%)
    - Enza = 12
    - Abi = 20
  - Low = 43 (57.3%%)
    - Enza = 25
    - Abi = 18
- Visceral mets
  - Yes = 15 (20%)
  - No = 60 (80%)
- Prior Chemotherapy = 34 (45.3%)
  - Abiraterone – 20
  - Enzalutamide - 14
- Abiraterone group
  - Prior Enzalutamide 9
- Enzalutamide group
  - Prior Abiraterone 2

**Response Rates:**

- PSA Response rate Whole cohort – 41 (54%)
  - Abiraterone – 39.5 % (15)
  - Enzalutamide -70.3% (26)

**Dose reduction**

- Whole cohort – 18 (24 %)
  - Abiraterone – 5 (13.2%)
  - Enzalutamide – 13 (35.1%)

**Dose interruption**

- Whole cohort – 16 (21.3) %
  - Abiraterone – 8 (21.1%)
  - Enzalutamide – 8 (21.6%)

**Median Follow up duration = 37 months**

***PSA PFS* Whole Cohort = 6 months**

| **Means and Medians for Survival Time** | | | | | | |
| --- | --- | --- | --- | --- | --- | --- |
| Mean^a^ | | | | Median | | |
| Estimate | Std. Error | 95% Confidence Interval | | Estimate | Std. Error | 95% Confidence Interval |
|  |  | Lower Bound | Upper Bound |  |  | Lower Bound |
| 10.463 | 1.399 | 7.722 | 13.204 | **6.000** | .787 | 4.457 |

| **Means and Medians for Survival Time** |
| --- |
| Median^a^ |
| 95% Confidence Interval |
| Upper Bound |
| 7.543 |

| a. Estimation is limited to the largest survival time if it is censored. |
| --- |


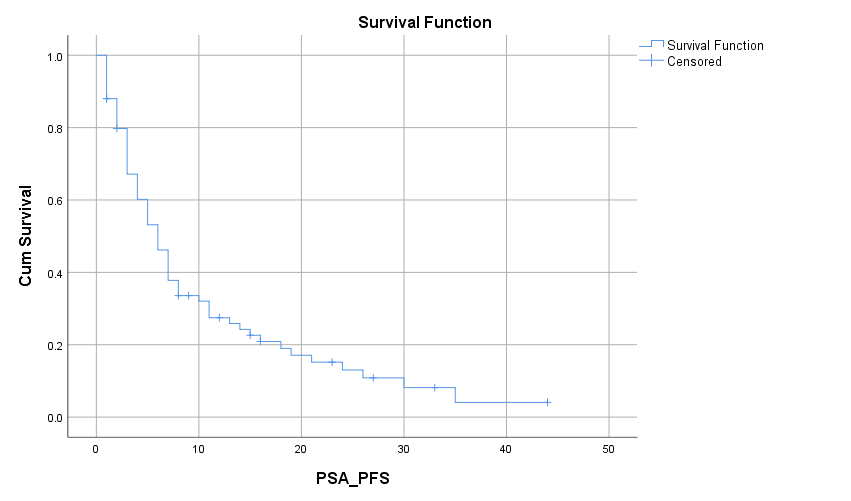


***PSA PFS***

**Abi (5 months) vs Enza (7 months)**

| **Means and Medians for Survival Time** | | | | | | |
| --- | --- | --- | --- | --- | --- | --- |
| Drug | Mean^a^ | | | | Median | |
|  | Estimate | Std. Error | 95% Confidence Interval | | Estimate | Std. Error |
|  |  |  | Lower Bound | Upper Bound |  |  |
| Abiraterone | 7.317 | 1.332 | 4.707 | 9.928 | **5.000** | .859 |
| Enzalutamide | 13.938 | 2.482 | 9.074 | 18.803 | **7.000** | 1.168 |
| Overall | 10.463 | 1.399 | 7.722 | 13.204 | 6.000 | .787 |

| **Means and Medians for Survival Time** | | |
| --- | --- | --- |
| Drug | Median^a^ | |
|  | 95% Confidence Interval | |
|  | Lower Bound | Upper Bound |
| Abiraterone | 3.317 | 6.683 |
| Enzalutamide | 4.710 | 9.290 |
| Overall | 4.457 | 7.543 |

| a. Estimation is limited to the largest survival time if it is censored. |
| --- |

| **Overall Comparisons** | | | |
| --- | --- | --- | --- |
|  | Chi-Square | df | Sig. |
| Log Rank (Mantel-Cox) | 5.259 | 1 | .022 |

| Test of equality of survival distributions for the different levels of Drug. |
| --- |


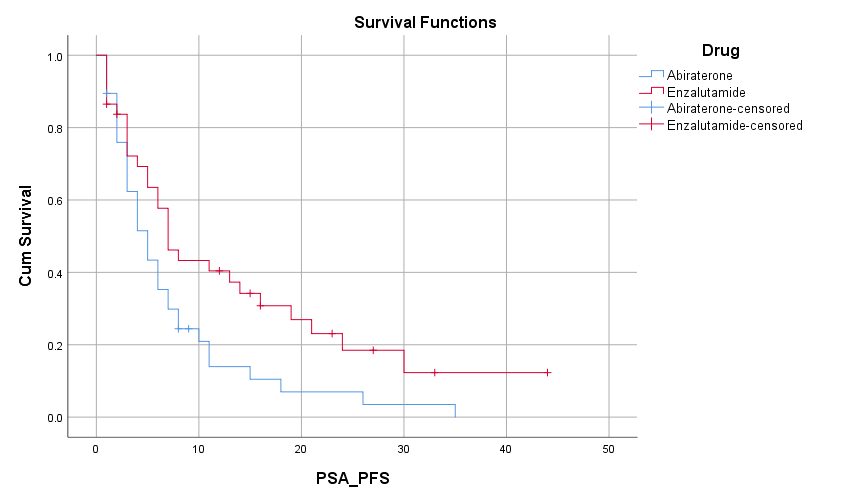


***PSA PFS***

**High Burden (4 months) vs Low burden (7 months)**

| **Means and Medians for Survival Time** | | | | | |
| --- | --- | --- | --- | --- | --- |
| Burden of Disease 1= High 2= Low | Mean^a^ | | | | Median |
|  | Estimate | Std. Error | 95% Confidence Interval | | Estimate |
|  |  |  | Lower Bound | Upper Bound |  |
| High | 6.169 | .907 | 4.391 | 7.947 | **4.000** |
| Low | 14.005 | 2.265 | 9.566 | 18.444 | **7.000** |
| Overall | 10.463 | 1.399 | 7.722 | 13.204 | 6.000 |

| **Means and Medians for Survival Time** | | | |
| --- | --- | --- | --- |
| Burden of Disease 1= High 2= Low | Median^a^ | | |
|  | Std. Error | 95% Confidence Interval | |
|  |  | Lower Bound | Upper Bound |
| High | 1.131 | 1.783 | 6.217 |
| Low | 2.467 | 2.165 | 11.835 |
| Overall | .787 | 4.457 | 7.543 |

| a. Estimation is limited to the largest survival time if it is censored. |
| --- |

| **Overall Comparisons** | | | |
| --- | --- | --- | --- |
|  | Chi-Square | df | Sig. |
| Log Rank (Mantel-Cox) | 8.487 | 1 | .004 |

| Test of equality of survival distributions for the different levels of Burden of Disease 1= High 2= Low. |
| --- |


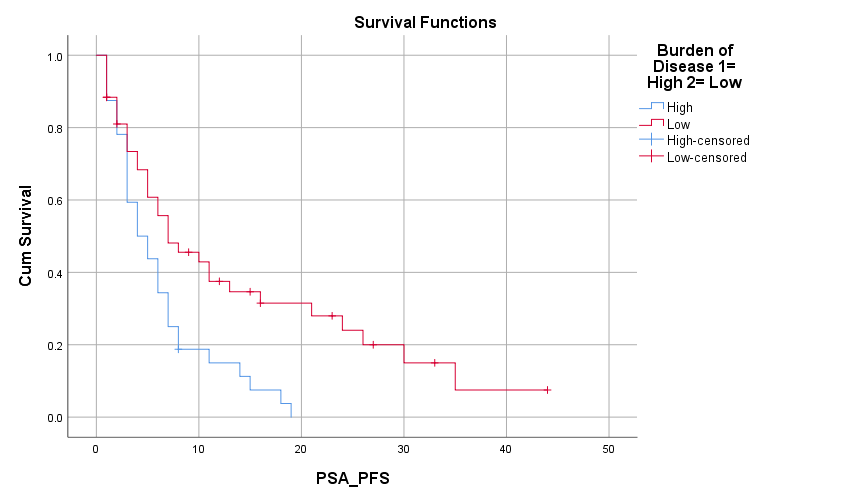


***PSA PFS***

**No prior chemotherapy (7 months) vs prior chemotherapy (4 months)**

| **Means and Medians for Survival Time** | | | | | | |
| --- | --- | --- | --- | --- | --- | --- |
| Prior Chemotherapy | Mean^a^ | | | | Median | |
|  | Estimate | Std. Error | 95% Confidence Interval | | Estimate | Std. Error |
|  |  |  | Lower Bound | Upper Bound |  |  |
| No prior chemotherapy | 11.532 | 1.863 | 7.881 | 15.183 | **7.000** | .896 |
| Post chemotherapy | 8.732 | 1.822 | 5.160 | 12.304 | **4.000** | .926 |
| Overall | 10.463 | 1.399 | 7.722 | 13.204 | 6.000 | .787 |

| **Means and Medians for Survival Time** | | |
| --- | --- | --- |
| Prior Chemotherapy | Median^a^ | |
|  | 95% Confidence Interval | |
|  | Lower Bound | Upper Bound |
| No prior chemotherapy | 5.243 | 8.757 |
| Post chemotherapy | 2.185 | 5.815 |
| Overall | 4.457 | 7.543 |

| a. Estimation is limited to the largest survival time if it is censored. |
| --- |

| **Overall Comparisons** | | | |
| --- | --- | --- | --- |
|  | Chi-Square | df | Sig. |
| Log Rank (Mantel-Cox) | 1.406 | 1 | .236 |

| Test of equality of survival distributions for the different levels of Prior Chemotherapy . |
| --- |


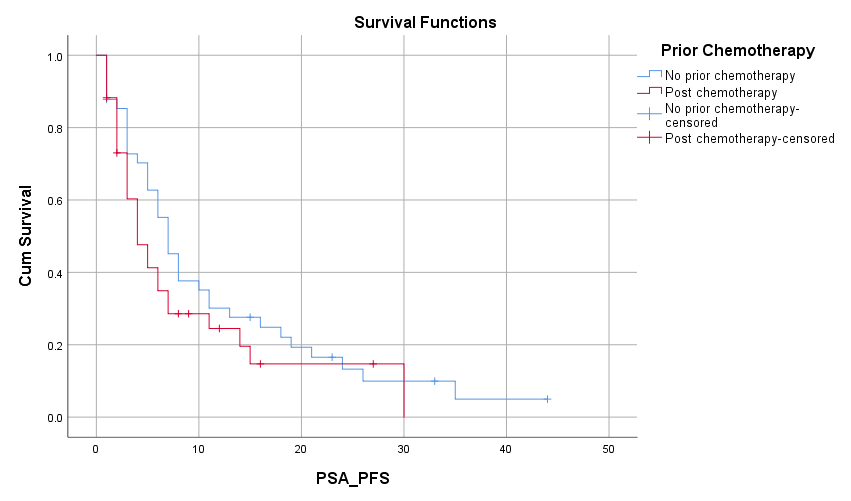


| **Means and Medians for Survival Time** | | | | | |
| --- | --- | --- | --- | --- | --- |
| Prior Chemotherapy | Drug | Median | | | |
|  |  | Estimate | Std. Error | 95% Confidence Interval | |
|  |  |  |  | Lower Bound | Upper Bound |
| No prior chemotherapy | Abiraterone | **7.000** | 1.346 | 4.363 | 9.637 |
|  | Enzalutamide | **7.000** | 1.597 | 3.870 | 10.130 |
|  | Overall | 7.000 | .896 | 5.243 | 8.757 |
| Post chemotherapy | Abiraterone | **4.000** | .533 | 2.955 | 5.045 |
|  | Enzalutamide | **7.000** | 1.657 | 3.751 | 10.249 |
|  | Overall | 4.000 | .926 | 2.185 | 5.815 |
| Overall | Overall | 6.000 | .787 | 4.457 | 7.543 |

| a. Estimation is limited to the largest survival time if it is censored. |
| --- |

| **Overall Comparisons^a^** | | | |
| --- | --- | --- | --- |
|  | Chi-Square | df | Sig. |
| Log Rank (Mantel-Cox) | 4.103 | 1 | .043 |

| Test of equality of survival distributions for the different levels of Drug.^a^ |
| --- |
| a. Adjusted for Prior Chemotherapy . |

**Stratum: PriorChemotherapy = No prior chemotherapy**


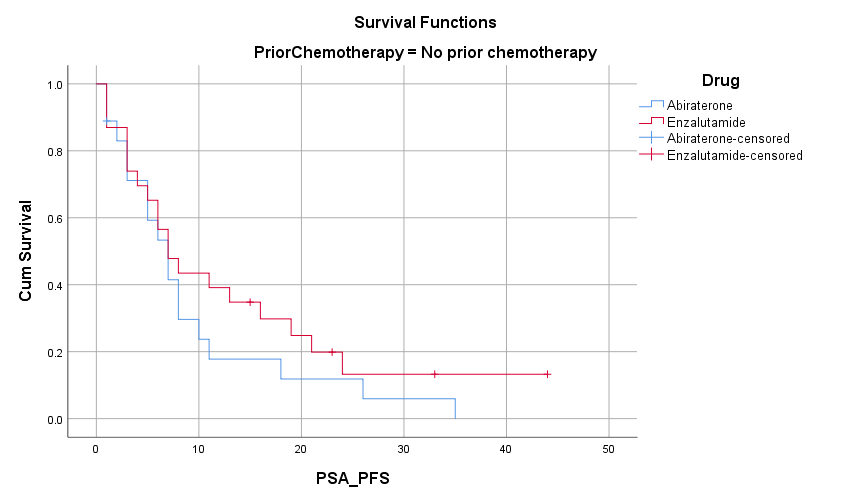


**Stratum: PriorChemotherapy = Post chemotherapy**


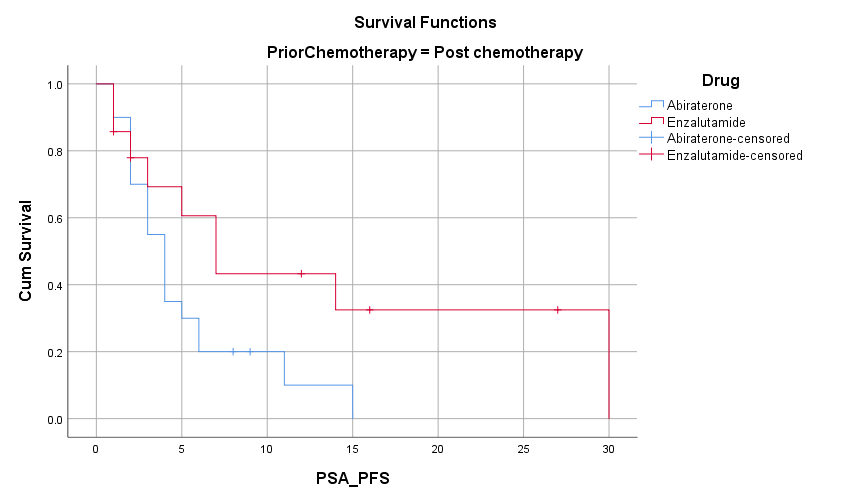


***Radiologic PFS* Whole Cohort = 7 months**

| **Means and Medians for Survival Time** | | | | | | |
| --- | --- | --- | --- | --- | --- | --- |
| Mean^a^ | | | | Median | | |
| Estimate | Std. Error | 95% Confidence Interval | | Estimate | Std. Error | 95% Confidence Interval |
|  |  | Lower Bound | Upper Bound |  |  | Lower Bound |
| 11.085 | 1.417 | 8.308 | 13.863 | 7.000 | .835 | 5.363 |

| **Means and Medians for Survival Time** |
| --- |
| Median^a^ |
| 95% Confidence Interval |
| Upper Bound |
| 8.637 |

| a. Estimation is limited to the largest survival time if it is censored. |
| --- |


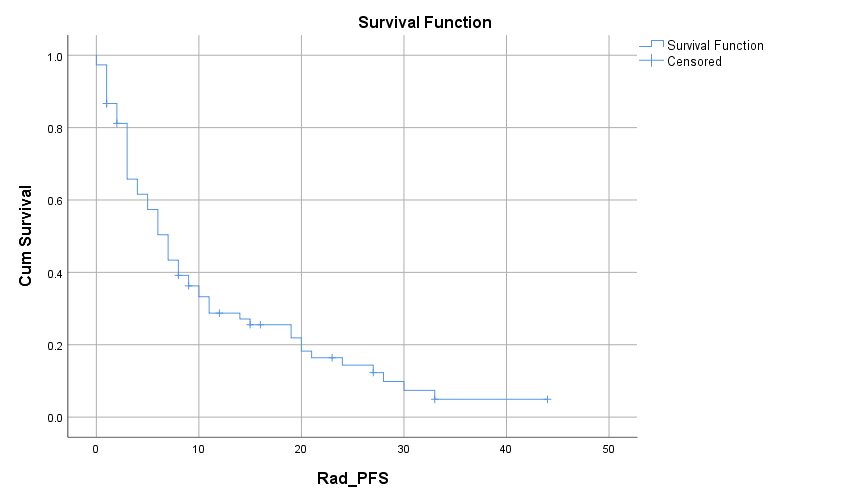


***Radiologic PFS***

**Abi (5 months) vs Enza (7 months)**

| **Means and Medians for Survival Time** | | | | | | |
| --- | --- | --- | --- | --- | --- | --- |
| Drug | Mean^a^ | | | | Median | |
|  | Estimate | Std. Error | 95% Confidence Interval | | Estimate | Std. Error |
|  |  |  | Lower Bound | Upper Bound |  |  |
| Abiraterone | 7.933 | 1.281 | 5.423 | 10.443 | 5.000 | 1.515 |
| Enzalutamide | 14.504 | 2.505 | 9.593 | 19.414 | 7.000 | 1.748 |
| Overall | 11.085 | 1.417 | 8.308 | 13.863 | 7.000 | .835 |

| **Means and Medians for Survival Time** | | |
| --- | --- | --- |
| Drug | Median^a^ | |
|  | 95% Confidence Interval | |
|  | Lower Bound | Upper Bound |
| Abiraterone | 2.030 | 7.970 |
| Enzalutamide | 3.573 | 10.427 |
| Overall | 5.363 | 8.637 |

| a. Estimation is limited to the largest survival time if it is censored. |
| --- |

| **Overall Comparisons** | | | |
| --- | --- | --- | --- |
|  | Chi-Square | df | Sig. |
| Log Rank (Mantel-Cox) | 4.416 | 1 | .036 |

| Test of equality of survival distributions for the different levels of Drug. |
| --- |


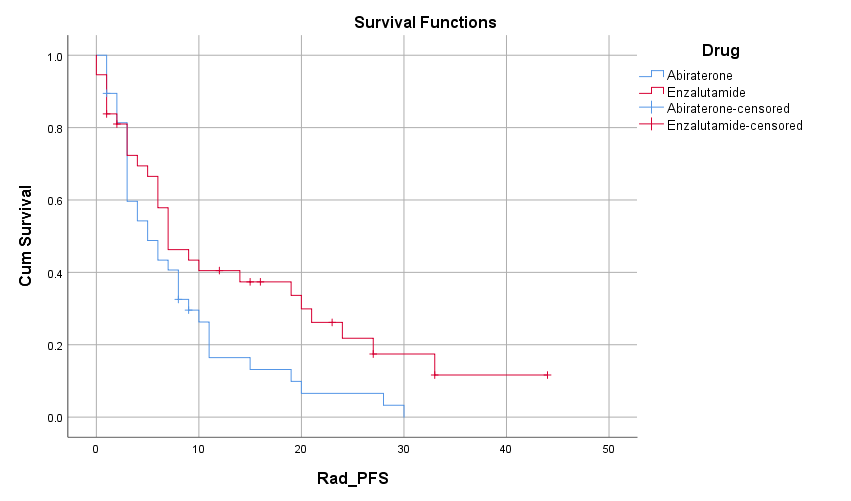


***Radiologic PFS***

**No prior chemotherapy (7 months) vs prior chemotherapy (6 months)**

| **Means and Medians for Survival Time** | | | | | | |
| --- | --- | --- | --- | --- | --- | --- |
| Prior Chemotherapy | Mean^a^ | | | | Median | |
|  | Estimate | Std. Error | 95% Confidence Interval | | Estimate | Std. Error |
|  |  |  | Lower Bound | Upper Bound |  |  |
| No prior chemotherapy | 11.538 | 1.910 | 7.795 | 15.281 | **7.000** | 1.047 |
| Post chemotherapy | 10.319 | 1.983 | 6.433 | 14.206 | **6.000** | 1.667 |
| Overall | 11.085 | 1.417 | 8.308 | 13.863 | 7.000 | .835 |

| **Means and Medians for Survival Time** | | |
| --- | --- | --- |
| Prior Chemotherapy | Median^a^ | |
|  | 95% Confidence Interval | |
|  | Lower Bound | Upper Bound |
| No prior chemotherapy | 4.947 | 9.053 |
| Post chemotherapy | 2.732 | 9.268 |
| Overall | 5.363 | 8.637 |

| a. Estimation is limited to the largest survival time if it is censored. |
| --- |

| **Overall Comparisons** | | | |
| --- | --- | --- | --- |
|  | Chi-Square | df | Sig. |
| Log Rank (Mantel-Cox) | .195 | 1 | .659 |

| Test of equality of survival distributions for the different levels of Prior Chemotherapy . |
| --- |


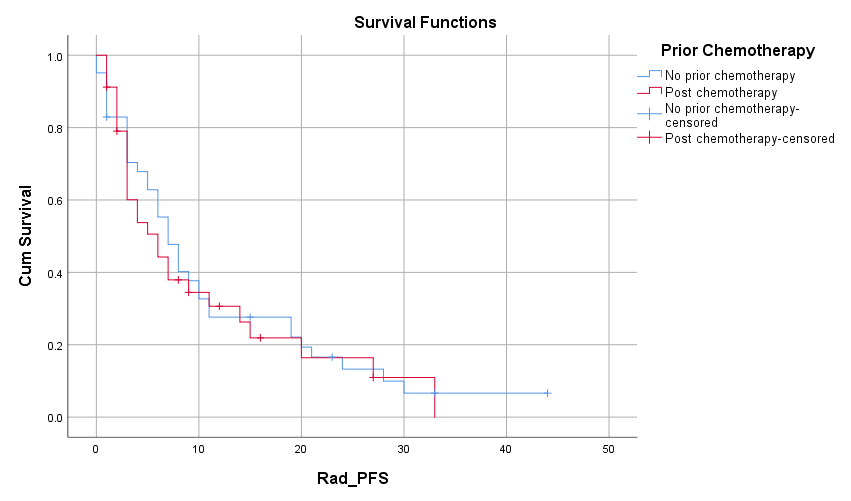


| **Means and Medians for Survival Time** | | | | | |
| --- | --- | --- | --- | --- | --- |
| Prior Chemotherapy | Drug | Median | | | |
|  |  | Estimate | Std. Error | 95% Confidence Interval | |
|  |  |  |  | Lower Bound | Upper Bound |
| No prior chemotherapy | Abiraterone | **8.000** | .673 | 6.681 | 9.319 |
|  | Enzalutamide | **6.000** | .958 | 4.122 | 7.878 |
|  | Overall | 7.000 | 1.047 | 4.947 | 9.053 |
| Post chemotherapy | Abiraterone | **3.000** | .639 | 1.748 | 4.252 |
|  | Enzalutamide | **14.000** | 5.501 | 3.218 | 24.782 |
|  | Overall | 6.000 | 1.667 | 2.732 | 9.268 |
| Overall | Overall | 7.000 | .835 | 5.363 | 8.637 |

| a. Estimation is limited to the largest survival time if it is censored. |
| --- |

| **Overall Comparisons^a^** | | | |
| --- | --- | --- | --- |
|  | Chi-Square | df | Sig. |
| Log Rank (Mantel-Cox) | 3.670 | 1 | .055 |

| Test of equality of survival distributions for the different levels of Drug.^a^ |
| --- |
| a. Adjusted for Prior Chemotherapy . |

**Stratum: PriorChemotherapy = No prior chemotherapy**


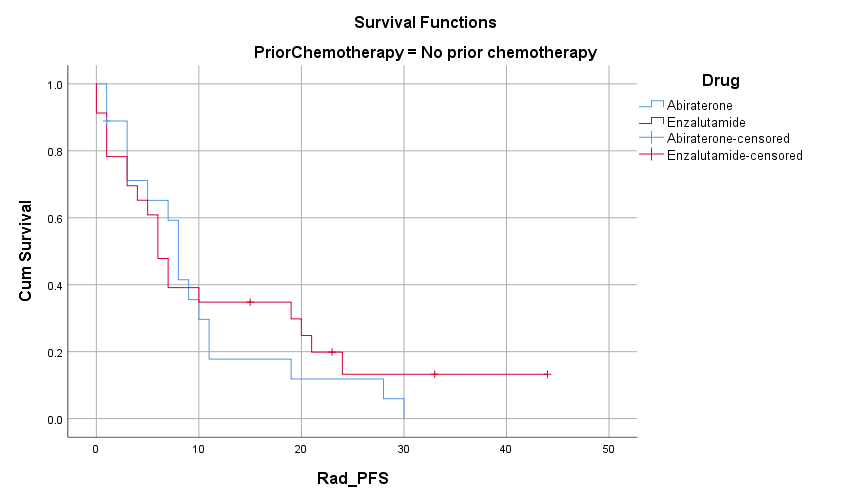


**Stratum: PriorChemotherapy = Post chemotherapy**


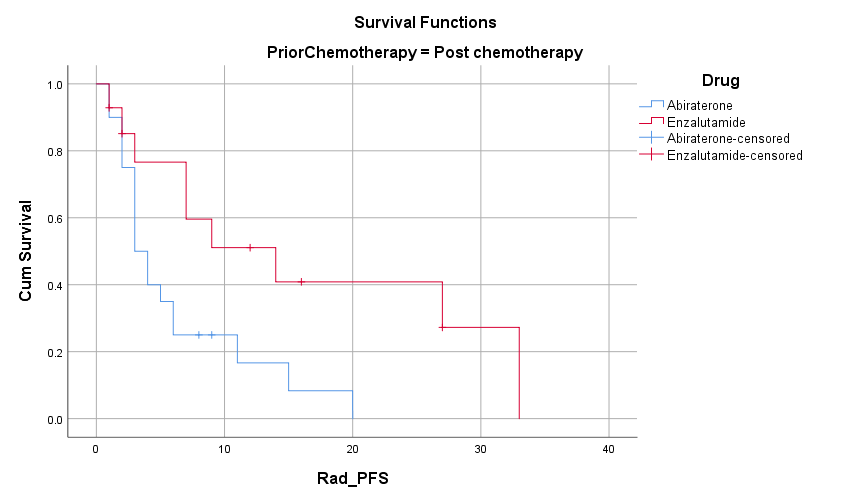


***Radiologic PFS***

**High Burden (6 months) vs Low burden (8 months)**

| **Means and Medians for Survival Time** | | | | | |
| --- | --- | --- | --- | --- | --- |
| Burden of Disease 1= High 2= Low | Mean^a^ | | | | Median |
|  | Estimate | Std. Error | 95% Confidence Interval | | Estimate |
|  |  |  | Lower Bound | Upper Bound |  |
| High | 7.719 | 1.209 | 5.349 | 10.088 | **6.000** |
| Low | 14.047 | 2.317 | 9.506 | 18.588 | **8.000** |
| Overall | 11.085 | 1.417 | 8.308 | 13.863 | 7.000 |

| **Means and Medians for Survival Time** | | | |
| --- | --- | --- | --- |
| Burden of Disease 1= High 2= Low | Median^a^ | | |
|  | Std. Error | 95% Confidence Interval | |
|  |  | Lower Bound | Upper Bound |
| High | 1.111 | 3.822 | 8.178 |
| Low | 1.742 | 4.586 | 11.414 |
| Overall | .835 | 5.363 | 8.637 |

| a. Estimation is limited to the largest survival time if it is censored. |
| --- |

| **Overall Comparisons** | | | |
| --- | --- | --- | --- |
|  | Chi-Square | df | Sig. |
| Log Rank (Mantel-Cox) | 5.072 | 1 | .024 |

| Test of equality of survival distributions for the different levels of Burden of Disease 1= High 2= Low. |
| --- |


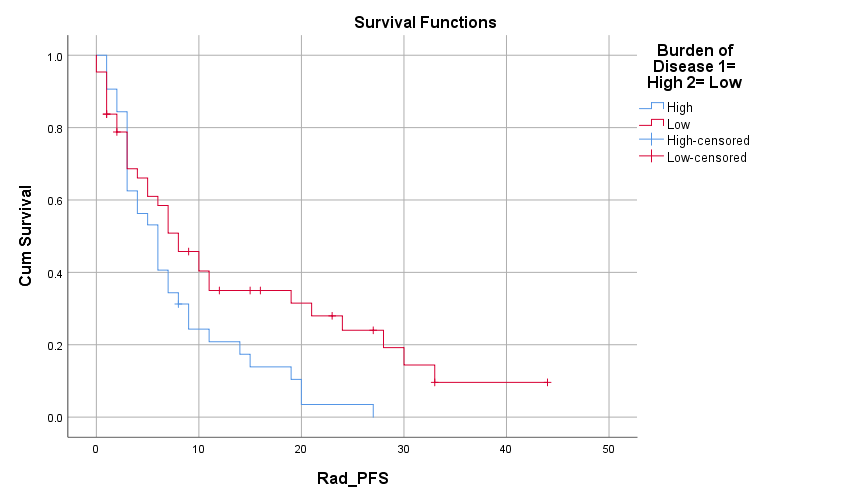


***Overall Survival* Whole Cohort = 24 months**

| **Means and Medians for Survival Time** | | | | | | |
| --- | --- | --- | --- | --- | --- | --- |
| Mean^a^ | | | | Median | | |
| Estimate | Std. Error | 95% Confidence Interval | | Estimate | Std. Error | 95% Confidence Interval |
|  |  | Lower Bound | Upper Bound |  |  | Lower Bound |
| 23.018 | 2.116 | 18.870 | 27.166 | **24.000** | 4.347 | 15.480 |

| **Means and Medians for Survival Time** |
| --- |
| Median^a^ |
| 95% Confidence Interval |
| Upper Bound |
| 32.520 |

| a. Estimation is limited to the largest survival time if it is censored. |
| --- |


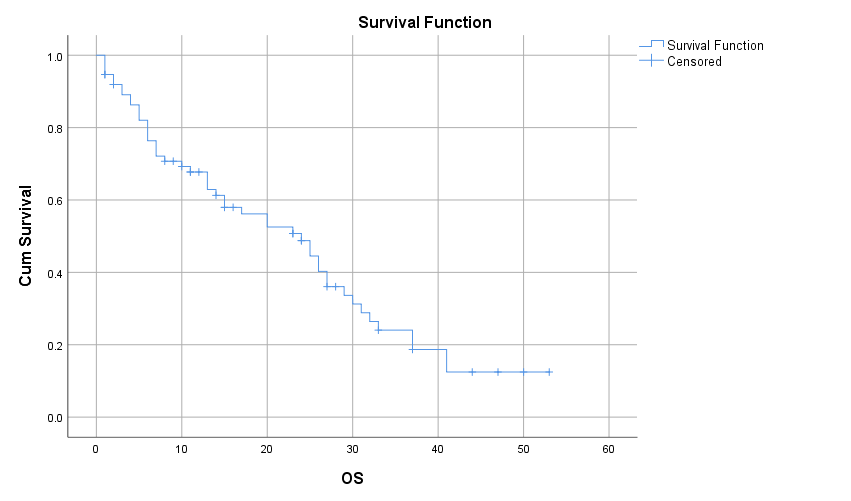


***Overall Survival***

**Abi (13 months) Vs Enza (30 months)**

| **Means and Medians for Survival Time** | | | | | | |
| --- | --- | --- | --- | --- | --- | --- |
| Drug | Mean^a^ | | | | Median | |
|  | Estimate | Std. Error | 95% Confidence Interval | | Estimate | Std. Error |
|  |  |  | Lower Bound | Upper Bound |  |  |
| Abiraterone | 16.197 | 2.455 | 11.385 | 21.009 | **13.000** | 2.586 |
| Enzalutamide | 29.790 | 2.978 | 23.953 | 35.627 | **30.000** | 2.611 |
| Overall | 23.018 | 2.116 | 18.870 | 27.166 | 24.000 | 4.347 |

| **Means and Medians for Survival Time** | | |
| --- | --- | --- |
| Drug | Median^a^ | |
|  | 95% Confidence Interval | |
|  | Lower Bound | Upper Bound |
| Abiraterone | 7.931 | 18.069 |
| Enzalutamide | 24.883 | 35.117 |
| Overall | 15.480 | 32.520 |

| a. Estimation is limited to the largest survival time if it is censored. |
| --- |

| **Overall Comparisons** | | | |
| --- | --- | --- | --- |
|  | Chi-Square | df | Sig. |
| Log Rank (Mantel-Cox) | 10.270 | 1 | .001 |

| Test of equality of survival distributions for the different levels of Drug. |
| --- |


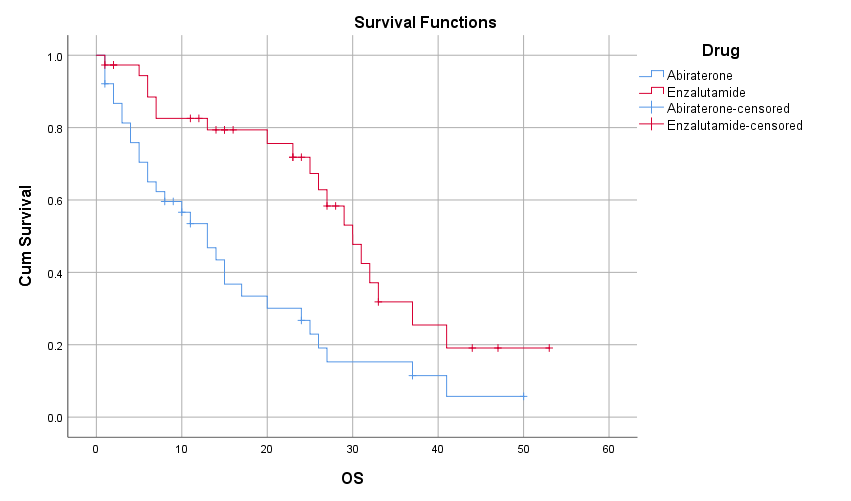


***Overall Survival***

**No prior chemotherapy (26 months) Vs prior chemotherapy (17 months)**

| **Means and Medians for Survival Time** | | | | | | |
| --- | --- | --- | --- | --- | --- | --- |
| Prior Chemotherapy | Mean^a^ | | | | Median | |
|  | Estimate | Std. Error | 95% Confidence Interval | | Estimate | Std. Error |
|  |  |  | Lower Bound | Upper Bound |  |  |
| No prior chemotherapy | 24.762 | 2.686 | 19.497 | 30.027 | **26.000** | 3.888 |
| Post chemotherapy | 19.900 | 3.102 | 13.819 | 25.981 | **17.000** | 7.010 |
| Overall | 23.018 | 2.116 | 18.870 | 27.166 | 24.000 | 4.347 |

| **Means and Medians for Survival Time** | | |
| --- | --- | --- |
| Prior Chemotherapy | Median^a^ | |
|  | 95% Confidence Interval | |
|  | Lower Bound | Upper Bound |
| No prior chemotherapy | 18.379 | 33.621 |
| Post chemotherapy | 3.261 | 30.739 |
| Overall | 15.480 | 32.520 |

| a. Estimation is limited to the largest survival time if it is censored. |
| --- |

| **Overall Comparisons** | | | |
| --- | --- | --- | --- |
|  | Chi-Square | df | Sig. |
| Log Rank (Mantel-Cox) | 1.037 | 1 | .308 |

| Test of equality of survival distributions for the different levels of Prior Chemotherapy . |
| --- |


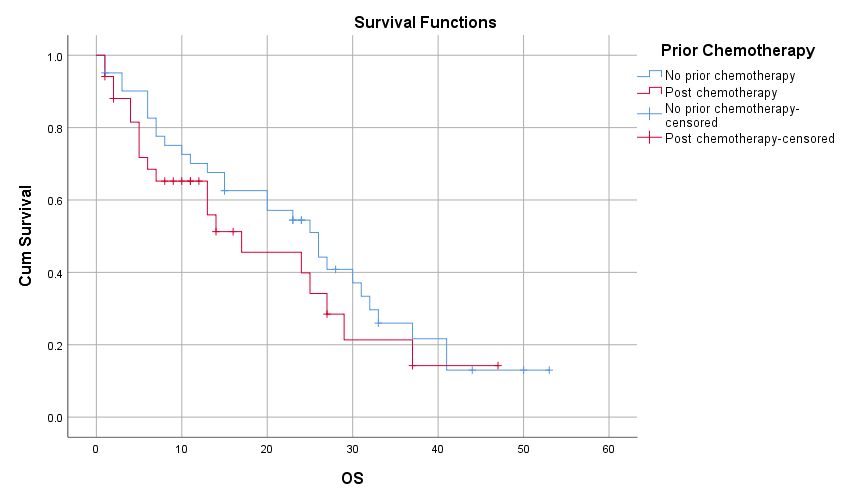


| **Means and Medians for Survival Time** | | | | | |
| --- | --- | --- | --- | --- | --- |
| Prior Chemotherapy | Drug | Median | | | |
|  |  | Estimate | Std. Error | 95% Confidence Interval | |
|  |  |  |  | Lower Bound | Upper Bound |
| No prior chemotherapy | Abiraterone | **15.000** | 2.699 | 9.710 | 20.290 |
|  | Enzalutamide | **30.000** | 3.402 | 23.331 | 36.669 |
|  | Overall | 26.000 | 3.888 | 18.379 | 33.621 |
| Post chemotherapy | Abiraterone | **7.000** | 5.870 | .000 | 18.505 |
|  | Enzalutamide | **29.000** | 3.949 | 21.261 | 36.739 |
|  | Overall | 17.000 | 7.010 | 3.261 | 30.739 |
| Overall | Overall | 24.000 | 4.347 | 15.480 | 32.520 |

| a. Estimation is limited to the largest survival time if it is censored. |
| --- |

| **Overall Comparisons^a^** | | | |
| --- | --- | --- | --- |
|  | Chi-Square | df | Sig. |
| Log Rank (Mantel-Cox) | 9.146 | 1 | .002 |

| Test of equality of survival distributions for the different levels of Drug.^a^ |
| --- |
| a. Adjusted for Prior Chemotherapy . |

**Stratum: PriorChemotherapy = No prior chemotherapy**


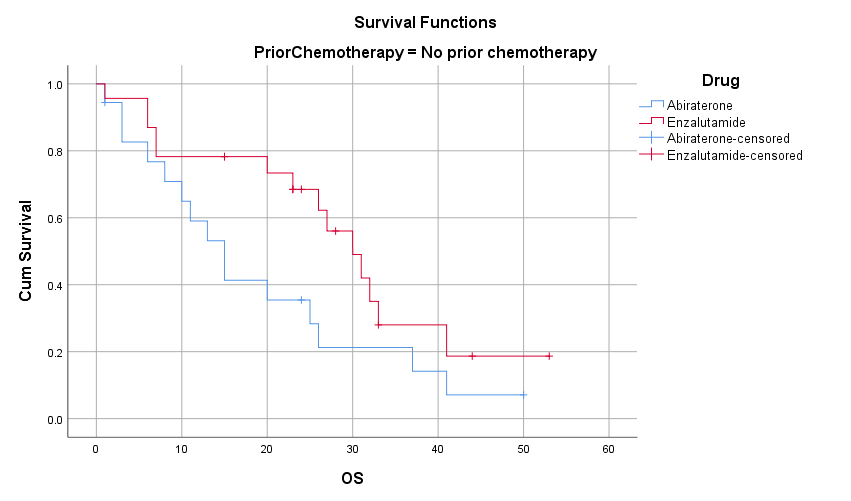


**Stratum: PriorChemotherapy = Post chemotherapy**


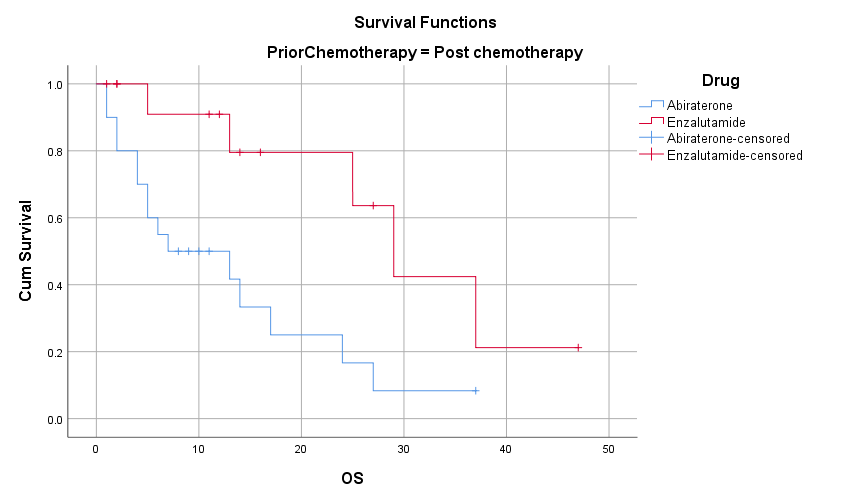


***Overall Survival***

**High burden (15 months) Vs Low burden (26 months)**

| **Means and Medians for Survival Time** | | | | | |
| --- | --- | --- | --- | --- | --- |
| Burden of Disease 1= High 2= Low | Mean^a^ | | | | Median |
|  | Estimate | Std. Error | 95% Confidence Interval | | Estimate |
|  |  |  | Lower Bound | Upper Bound |  |
| High | 19.274 | 3.006 | 13.383 | 25.166 | **15.000** |
| Low | 25.694 | 2.743 | 20.317 | 31.071 | **26.000** |
| Overall | 23.018 | 2.116 | 18.870 | 27.166 | 24.000 |

| **Means and Medians for Survival Time** | | | |
| --- | --- | --- | --- |
| Burden of Disease 1= High 2= Low | Median^a^ | | |
|  | Std. Error | 95% Confidence Interval | |
|  |  | Lower Bound | Upper Bound |
| High | 4.656 | 5.874 | 24.126 |
| Low | 1.765 | 22.540 | 29.460 |
| Overall | 4.347 | 15.480 | 32.520 |

| a. Estimation is limited to the largest survival time if it is censored. |
| --- |

| **Overall Comparisons** | | | |
| --- | --- | --- | --- |
|  | Chi-Square | df | Sig. |
| Log Rank (Mantel-Cox) | 2.558 | 1 | .110 |

| Test of equality of survival distributions for the different levels of Burden of Disease 1= High 2= Low. |
| --- |


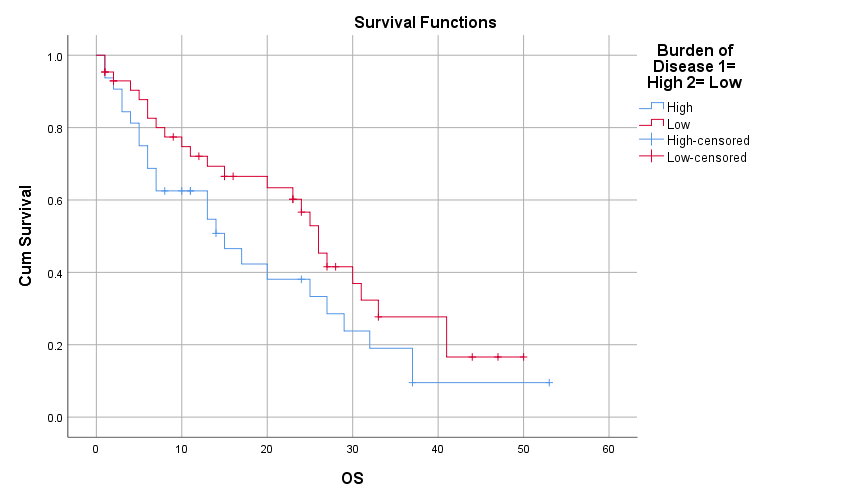

Supplement: Supplementary file 1 [file DataSheet_1.docx]
