# Supplementary material for: Real-World Data on Outcomes in Metastatic Castrate-Resistant Prostate Cancer Patients Treated With Abiraterone or Enzalutamide: A Regional Experience
Source: Front Oncol. 2021 Jun 18;11:656146. doi: 10.3389/fonc.2021.656146 (PMC8249852; doi:10.3389/fonc.2021.656146)
Supplement: Supplementary file 2 [file Presentation_1.pptx]

## Slide 1
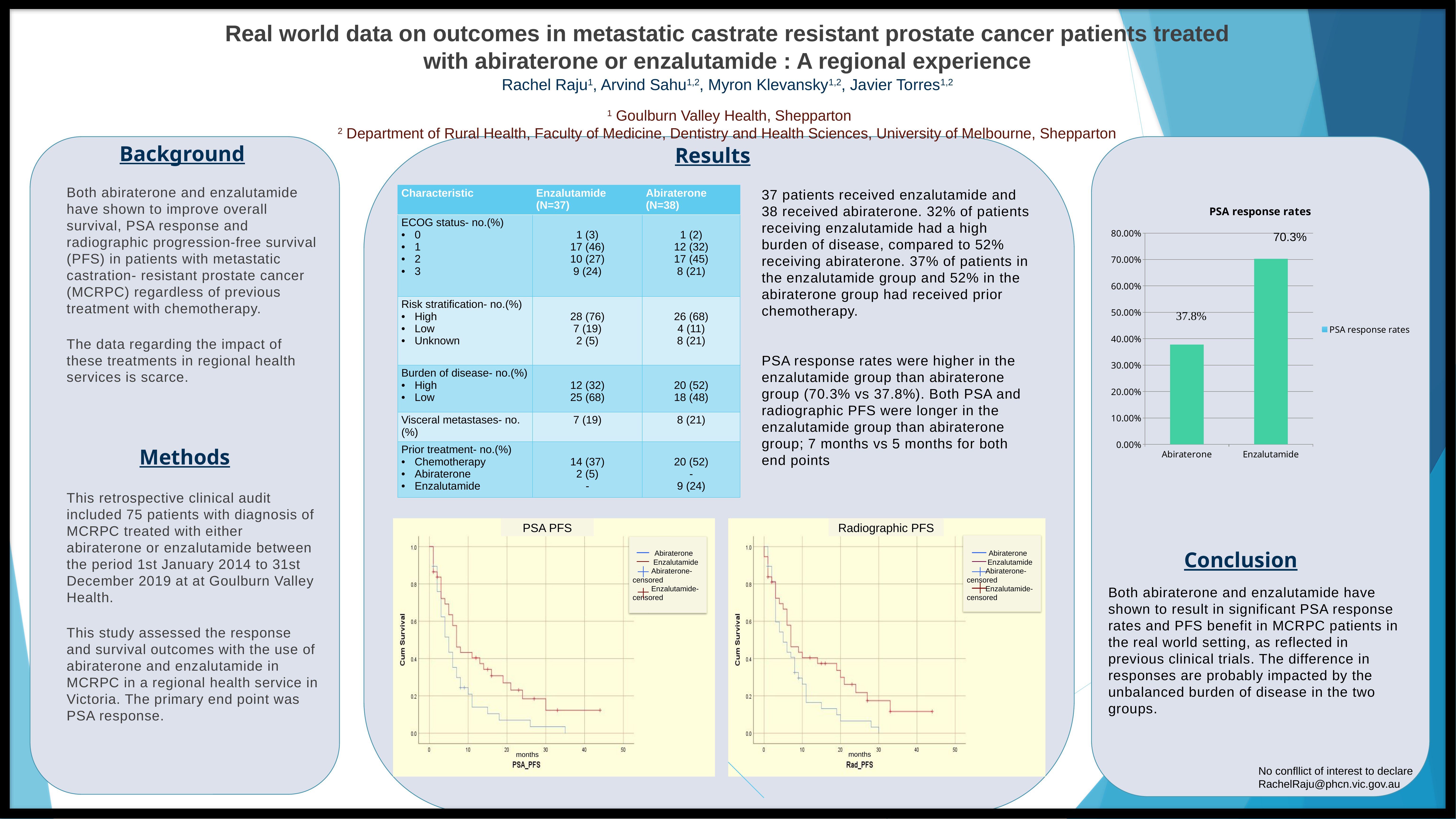

Real world data on outcomes in metastatic castrate resistant prostate cancer patients treated with abiraterone or enzalutamide : A regional experience
Rachel Raju1, Arvind Sahu1,2, Myron Klevansky1,2, Javier Torres1,2
1 Goulburn Valley Health, Shepparton
2 Department of Rural Health, Faculty of Medicine, Dentistry and Health Sciences, University of Melbourne, Shepparton
Results
Background
Both abiraterone and enzalutamide have shown to improve overall survival, PSA response and radiographic progression-free survival (PFS) in patients with metastatic castration- resistant prostate cancer (MCRPC) regardless of previous treatment with chemotherapy.
The data regarding the impact of these treatments in regional health services is scarce.
37 patients received enzalutamide and 38 received abiraterone. 32% of patients receiving enzalutamide had a high burden of disease, compared to 52% receiving abiraterone. 37% of patients in the enzalutamide group and 52% in the abiraterone group had received prior chemotherapy.
PSA response rates were higher in the enzalutamide group than abiraterone group (70.3% vs 37.8%). Both PSA and radiographic PFS were longer in the enzalutamide group than abiraterone group; 7 months vs 5 months for both end points
..
| Characteristic | Enzalutamide (N=37) | Abiraterone (N=38) |
| --- | --- | --- |
| ECOG status- no.(%) 0 1 2 3 | 1 (3) 17 (46) 10 (27) 9 (24) | 1 (2) 12 (32) 17 (45) 8 (21) |
| Risk stratification- no.(%) High Low Unknown | 28 (76) 7 (19) 2 (5) | 26 (68) 4 (11) 8 (21) |
| Burden of disease- no.(%) High Low | 12 (32) 25 (68) | 20 (52) 18 (48) |
| Visceral metastases- no.(%) | 7 (19) | 8 (21) |
| Prior treatment- no.(%) Chemotherapy Abiraterone Enzalutamide | 14 (37) 2 (5) - | 20 (52) - 9 (24) |
### Chart:
| Category | PSA response rates |
|---|---|
| Abiraterone | 0.378 |
| Enzalutamide | 0.703 |70.3%
Methods
This retrospective clinical audit included 75 patients with diagnosis of MCRPC treated with either abiraterone or enzalutamide between the period 1st January 2014 to 31st December 2019 at at Goulburn Valley Health.
This study assessed the response and survival outcomes with the use of abiraterone and enzalutamide in MCRPC in a regional health service in Victoria. The primary end point was PSA response.
PSA PFS
Radiographic PFS
Conclusion
 Abiraterone
 Enzalutamide
 Abiraterone- censored
 Enzalutamide- censored
 Abiraterone
 Enzalutamide
 Abiraterone- censored
 Enzalutamide- censored
Both abiraterone and enzalutamide have shown to result in significant PSA response rates and PFS benefit in MCRPC patients in the real world setting, as reflected in previous clinical trials. The difference in responses are probably impacted by the unbalanced burden of disease in the two groups.
months
months
No confllict of interest to declare
RachelRaju@phcn.vic.gov.au
